# Importance of SNP Dependency Correction and Association Integration for Gene Set Analysis in Genome-Wide Association Studies

**DOI:** 10.3389/fgene.2021.767358

**Published:** 2021-12-09

**Authors:** Michal Marczyk, Agnieszka Macioszek, Joanna Tobiasz, Joanna Polanska, Joanna Zyla

**Affiliations:** ^1^ Department of Data Science and Engineering, Silesian University of Technology, Gliwice, Poland; ^2^ Yale Cancer Center, Yale School of Medicine, New Haven, CT, United States

**Keywords:** gene set analysis, genome-wide association study, statistical integration, single-nucleotide polymorphism, linkage disequilibrium correction

## Abstract

A typical genome-wide association study (GWAS) analyzes millions of single-nucleotide polymorphisms (SNPs), several of which are in a region of the same gene. To conduct gene set analysis (GSA), information from SNPs needs to be unified at the gene level. A widely used practice is to use only the most relevant SNP per gene; however, there are other methods of integration that could be applied here. Also, the problem of nonrandom association of alleles at two or more loci is often neglected. Here, we tested the impact of incorporation of different integrations and linkage disequilibrium (LD) correction on the performance of several GSA methods. Matched normal and breast cancer samples from The Cancer Genome Atlas database were used to evaluate the performance of six GSA algorithms: Coincident Extreme Ranks in Numerical Observations (CERNO), Gene Set Enrichment Analysis (GSEA), GSEA-SNP, improved GSEA for GWAS (i-GSEA4GWAS), Meta-Analysis Gene-set Enrichment of variaNT Associations (MAGENTA), and Over-Representation Analysis (ORA). Association of SNPs to phenotype was calculated using modified McNemar’s test. Results for SNPs mapped to the same gene were integrated using Fisher and Stouffer methods and compared with the minimum *p*-value method. Four common measures were used to quantify the performance of all combinations of methods. Results of GSA analysis on GWAS were compared to the one performed on gene expression data. Comparing all evaluation metrics across different GSA algorithms, integrations, and LD correction, we highlighted CERNO, and MAGENTA with Stouffer as the most efficient. Applying LD correction increased prioritization and specificity of enrichment outcomes for all tested algorithms. When Fisher or Stouffer were used with LD, sensitivity and reproducibility were also better. Using any integration method was beneficial in comparison with a minimum *p*-value method in specific combinations. The correlation between GSA results from genomic and transcriptomic level was the highest when Stouffer integration was combined with LD correction. We thoroughly evaluated different approaches to GSA in GWAS in terms of performance to guide others to select the most effective combinations. We showed that LD correction and Stouffer integration could increase the performance of enrichment analysis and encourage the usage of these techniques.

## Introduction

Genome-wide association study (GWAS) is a high-throughput molecular biology technique, which gives insight into understanding the relation of single-nucleotide polymorphism (SNP) frequency and other types of genetic variations with particular traits. In recent years, GWAS reveals plenty of genetic locations related to common diseases, e.g., type 2 diabetes (Billings and Florez, 2010), Alzheimer disease (Marioni et al., 2018), or many types of cancer (Sud et al., 2017). Despite the promising outcomes, the biological functions of many genetic variation loci remain unclear, and the genetic mechanisms of phenotypes are not systematically explained. Yet, the GWAS is still an important tool used to understand the biological mechanisms of different diseases (Wijmenga and Zhernakova, 2018). One of the bioinformatic techniques, which can extend the amount of information from single genetic variations and their impact on the biological systems, is gene set analysis (GSA), and the importance of such a solution has been recently noticed (Wang et al., 2007; Holden et al., 2008; Hirschhorn, 2009; Zhang et al., 2010; Weng et al., 2011; de Leeuw et al., 2015; Mei et al., 2016; Sud et al., 2017; Yoon et al., 2018; Maleki et al., 2020).

The GSA allows summarizing the results of association with phenotype from individual gene level to gene set level, also known as pathway level. Using this concept, it is possible to detect the aggregated impact of multiple genes on phenotype, even when the individual gene has moderate or small effect on the investigated trait. In addition, applying GSA increases understanding of changes observed in complex biological mechanisms under various conditions. Within the last decade of gene set analysis method development, many algorithms were introduced [just to mention a few: GSEA (Subramanian et al., 2005), PADOG (Tarca et al., 2012), SPIA (Tarca et al., 2009) or LEGO (Dong et al., 2016)] and can be classified by their generation (Khatri et al., 2012; Zyla et al., 2017), hypothesis tested (Maciejewski, 2014), or application for a particular omics platform (Das et al., 2020). First, algorithms were created to analyze the gene expression data from microarray experiments, but with rapid advancement in molecular biology techniques, they became widely applied in other omics, resulting in growth of bioinformatic tools, which perform multi-omics gene set analysis (Canzler and Hackermüller, 2020; Kaspi and Ziemann, 2020). Application of GSA techniques to dissimilar omics data is associated with different problems. In the analysis of GWAS results, the key issue is how to transform the observed genetic variation into gene level. One of the most used techniques is to choose the SNP with the strongest association (minimum *p*-value) to represent a gene (Wang et al., 2007; Zhang et al., 2010). The minimum *p*-value approach may not be an optimal solution as it favors long genes with many SNPs measured, where obtaining stronger association is more likely compared with shorter ones. Thus, some adjustments were introduced to correct this effect, e.g., adaptive *p*-value combination of *p*-values (Yu et al., 2009), selecting representative SNPs for each gene (Weng et al., 2011), or correction of smallest *p*-value due to some factors, like no. of SNPs per kb, gene size, and linkage disequilibrium units per kb (Segrè et al., 2010). Other aggregation techniques, like Fisher integration, second minimum *p*-value, or application of Simes’ *p*-value adjusted for the number of SNPs were also proposed (Mei et al., 2016). However, the authors applied those approaches only to the oldest gene set analysis method based on hypergeometric test [over-representation analysis (ORA)]. Also, they performed only basic evaluation, concentrating mostly on detecting target pathways for the analyzed dataset without looking at false positives. Recently, a new method of GSA in GWAS was introduced and compared with other methods by Type 1 error control and statistical power (Yoon et al., 2018; Sun et al., 2019), but without testing different integration methods or SNP dependency correction. Finally, there are solutions where the problem of aggregation from genome to transcriptome level was neglected, e.g., MAGMA (de Leeuw et al., 2015) or GSEA-SNP (Holden et al., 2008).

Even though GSA methods have been used for over a decade in omics data analysis, there still exist many challenges in this research field (Maleki et al., 2020). The knowledge about GSA algorithm efficiency was widely updated in several publications (Mitrea et al., 2013; Tarca et al., 2013; Maleki et al., 2019; Nguyen et al., 2019; Zyla et al., 2019; Geistlinger et al., 2021; Xie et al., 2021). Yet, those studies concentrated on enrichment methods dedicated to gene expression data measured with microarrays or RNA sequencing (RNASeq) technologies, and the overall performance of GSA algorithms in other omics is still not known. In this work, we focused on two major difficulties that occur during applying GSA in GWAS studies. The first goal of the study was to test the impact of aggregation of phenotype association test results from SNP to gene level, which is then transformed to gene set level. For this purpose, three statistical integration techniques were tested in a variety of GSA algorithms. The second goal was to investigate the impact of linkage disequilibrium (LD) control in the process of SNP information aggregation. These two GSA extensions were tested in combination with six gene set analysis methods. Each tested combination of algorithms was evaluated in terms of sensitivity, specificity, prioritization, and reproducibility of gene set analysis. Furthermore, the relation of GSA GWAS results to those obtained on gene expression data was investigated using the same collection of patient samples. Finally, all tested GSA algorithms, integration techniques, and LD correction were implemented in R package *intGSASNP* (integrative GSA for SNP).

## Materials and Methods

### Data

Data from Affymetrix Genome-Wide Human SNP Array 6.0 platform served for SNP genotyping. Affymetrix SNP 6.0 microarrays include over 906,600 SNPs and over 946,000 probes for copy number variation detection (Affymetrix, 2021). All files are part of The Cancer Genome Atlas (TCGA) Breast Invasive Carcinoma collection (Berger et al., 2018) and were downloaded in CEL format from the Genomic Data Commons (GDC) Legacy Archive. Only white female breast cancer patients were selected for the study. For all individuals, data for both primary tumors and solid normal tissues were available.

Subsequently, Illumina paired-end RNA sequencing data were used for the same patients and the same samples (both tumor and normal) as in the case of SNP genotyping. All data files were downloaded from GDC Data Portal as HTSeq-counts. GDC previously preprocessed raw sequencing data according to the bioinformatics pipeline available from GDC Documentation (NCI Genomic Data Commons, 2021).

Consequently, 83 white females were considered. For all of them, RNASeq and SNP microarray results were available for both tumor and normal tissue fresh frozen specimens, which summed up to 166 samples. Hence, all individuals were matched in terms of sample and experiment types. Specimens were collected at five different centers (tissue source sites) participating in TCGA Breast Invasive Carcinoma project. All patients were labeled with a breast cancer subtype as previously described (Marczyk et al., 2019; Marczyk et al., 2020). The summary of breast cancer subtypes, source center, and patient ethnicity is presented in Supplementary Table S1.

### Single-Nucleotide Polymorphism Data Analysis

For each genotyped SNP, genome location and relation to transcriptomic function were mapped using the ENSEMBL human genome database, v80 (May 2015; http://may2015.archive.ensembl.org; *biomaRt* R package) (Cunningham et al., 2015). During the process of quality control, multiple SNPs were filtered out due to minor allele frequency (MAF; lower than 5% removed) and Hardy–Weinberg equilibrium (HWE; *p*-value <0.05). Next, only SNPs that are located within the range of 5 kb upstream and 5 kb downstream of the gene were selected. The selected boundary is much narrower in comparison with other studies [e.g., (Segrè et al., 2010; Zhang et al., 2010)], but here, we wanted to reflect the strongest association to possible changes in gene expression. These steps reduced the initial number of SNPs from 905,176 to 240,799. Finally, to compute the association between genotypes and phenotype (breast cancer *vs.* healthy tissue) under genotype genetic model (AA/AB/BB) with the paired design, the multinomial exact test (extension of McNemar’s test) was performed with 100,000 Monte Carlo permutations using *rcompanion* R package (Mangiafico, 2016). This method does not allow introducing additional covariates in the analysis. As the collected samples come from white females only, the distributions of other biases between healthy and cancer tissue samples are the same due to the paired design of the experiment. Thus, the calculated model was not adjusted for other covariates.

In most cases, to perform GSA using SNP-level data, a single value per gene is needed. Thus, association results for SNPs within the same transcript need to be integrated into one representative value. Three different techniques for test result integration were applied. Currently, the most common method in GSA GWAS is to take the minimum *p*-value for SNP *i*, which falls within the gene *g* boundaries:

$$p-value_{\mathrm{gene}}=\underset{i\in g}{\min}\left\{p-value_{SNP}\right\}$$ (1)

The second integration technique evaluated here was Fisher’s probability integration (Fisher, 1925), which calculates the sum of the natural logarithm from *k* SNP *p*-values, which fall within the same gene *g* boundaries:

$$F_{\mathrm{gene}}=-2\overset{k}{\underset{i=1}{\sum }}\ln\left(p-value_{i}\right)\;∼\;\chi_{\left(2k\right)}^{2}$$ (2)

The calculated F statistic per gene, *F*_*gene*_, follows chi^2^ distribution with 2*k degrees of freedom.

The last statistical integration approach used was the Stouffer method, also known as z-transformation-based integration (Stouffer, 1949). For *k* SNPs, which fall within the same gene *g* boundaries, *Z*_*i*_ statistic is first calculated using inverse normal cumulative distribution function ( $\phi^{-1}$ ) for each *i*-th SNP. Then the integrated Z statistic per gene, *Z*_*gene*_, which follows standard normal distribution is calculated.

$$Z_{i}=\phi^{-1}\left(p-value_{i}\right)$$ (3)

$$Z_{\mathrm{gene}}=\frac{\sum_{i=1}^{k}Z_{i}}{\sqrt{k}}\;∼\;N\left(0,1\right)$$ (4)

Next, for the integrated *p*-values, the dependency correction due to LD was applied. The commonly used approach for LD correction requires calculations of *r* ^2^ or D′ score. Here, the modification of Dunn–Sidak correction for multiple testing was used instead. As was shown in Saccone et al. (2006), approximately 50% of the SNPs within chromosomes are in high LD; thus, the exponent of Dunn–Sidak was modified as follows:

$$p-value_{corr}=1-{\left(1-\;p-value_{gene}\right)}^{\frac{k+1}{2}}$$ (5)

where *k* is the number of SNPs located within gene *g.* This method of introducing LD correction was proposed by Saccone et al. (2007) and allows for running enrichment analysis even for very limited genotyping data consisting only of two elements: SNP rs number and the result of the association test. Moreover, it was shown that the method is comparable, or slightly better than the regression method of GWAS integration *p*-value with correction due to SNPs per kb, gene size, recombination hotspots, linkage disequilibrium units per kb, or genetic distance (Segrè et al., 2010). Each integration approach with or without dependency correction was tested in terms of effectiveness in GSA in GWAS.

### Enrichment Algorithms for Single-Nucleotide Polymorphism Data

Several GSA algorithms dedicated to GWAS are based on *p*-value integration to move from SNP to transcriptome level (Das et al., 2020). From this group, the algorithms based on the Gene Set Enrichment Analysis (GSEA) method, mostly used in transcriptomic analysis (Subramanian et al., 2005), were selected.

The basic concept of GSEA is to estimate the enrichment score (ES) by calculating maximum absolute deviation between *P*_*hit*_ (normalized metric of genes within gene set *S*) and *P*_*miss*_ (normalized metric for genes outside gene set *S*). The ES distribution is calculated for *j*-th gene *g* in gene set *S* at the *i*-th position by modified Smirnov–Kolmogorov statistic using the following formulas:

$$P_{hit}\left(S,i\right)=\sum_{\begin{array}{l} g_{j}\in S\\ j\leq i \end{array}}\frac{\left|r_{j}\right|}{N_{R}}$$ (6)

$$N_{R}=\sum_{\begin{array}{l} g_{j}\in S \end{array}}\left|r_{j}\right|$$ (7)

$$P_{miss}\left(S,i\right)=\sum_{\begin{array}{l} g_{j}\notin S\\ j\leq i \end{array}}\frac{1}{\left(N-N_{H}\right)}$$ (8)

Here, as a rank *r*, the negative value of base 10 logarithm of *p*-value was taken [−log10(*p*-value_gene_)]. *N* is the total number of genes, and *N*_*H*_ represents the number of genes in the gene set *S*, and *N*_*R*_ is the sum of ranks of genes within gene set *S*.

The next algorithm used was GSEA-SNP (Holden et al., 2008), which is a simple modification of the standard GSEA approach. In this method, instead of integrating SNP association to transcriptomic level, the *p*-values of all SNPs are taken to calculate rank *r* parameter [−log10(*p*-value_SNP_)]. Moreover, in GSEA-SNP, the SNP label permutation test is performed to assess significance of each gene set, while for GSEA, the gene label permutation is applied. In both GSEA and GSEA-SNP, the ES metric is adjusted for variation in gene set size by dividing the observed *ES* by the mean of permutated ES with the same direction giving normalized enrichment score (NES).

The third algorithm was *i*-GSEA4GWAS (improved GSEA for GWAS) (Zhang et al., 2010). This method has two main modifications compared with the standard GSEA: 1) Instead of gene label permutation, the SNP label permutation is performed, and then integration of *p*-values from SNP association test is executed. 2) The NES is substituted by significance proportion-based enrichment score (SPES). The SPES is multiplication of ES by ratio *k/K*, where *k* is the proportion of significant genes (mapped to 5% of the top SNPs) of the gene set *S*, and *K* is the proportion of significant genes (mapped to 5% of the top SNPs) of the total genes in the study (Zhang et al., 2010). According to the authors, SPES emphasizes the total significance coming from a high proportion of significant genes.

The fourth algorithm was MAGENTA (Meta-Analysis Gene-set Enrichment of variaNT Associations) (Segrè et al., 2010), where gene set significance is estimated as follows: 1) *p*-Values from SNP to gene level were integrated. 2) For each gene set, the number of gene *p*-values within a gene set lower than the cut-off (leading edge fraction) was calculated. The cutoff is a *p*-value of a specific percentile of all gene *p*-values (here set to the 75th percentile and marked as MAGENTA75), 3) to calculate the distribution of leading edge fraction with the permutation approach. In each permutation, the mock gene set is drawn as its leading edge fraction is collected. Finally, to assess the gene set significance, the number of permutation leading edge fractions equal or larger than the observed one for a particular gene set is estimated and divided by the number of permutations. All algorithms described above are modifications of GSEA approach and test *competitive* null hypothesis.

Two other algorithms were added to this list: ORA (over-representation analysis) (Tavazoie et al., 1999) and CERNO (Coincident Extreme Ranks in Numerical Observations) algorithm (Zyla et al., 2019). Both are designed for transcriptome data analysis but can be easily used in GWAS problems. ORA is the first-generation method, which estimates gene set significance via hypergeometric test using information about the number of differentially expressed genes (DEGs) and background genes within and outside the gene set. The CERNO method ranks genes from 1 to *N* (total analyzed genes), where rank 1 is given to the gene with the lowest *p*-value (here *p*-value from integration of SNP association). Next, the given ranks are divided by *N*, and the Fisher probability integration is performed for all genes within the gene set.

### RNASeq Data Analysis

Genes that were not represented in SNP data or with no counts within all samples were removed prior to analysis (15,924 genes left). *DESeq2* R package (Love et al., 2014) was used to find genes with different expressions between normal and cancer samples, including the paired nature of the data. Pathway enrichment analysis with the GSEA method was performed using the *fgsea* package in R (Korotkevich et al., 2019) on the same set of pathways used in SNP data analysis. Test statistic from the *DESEq2* package was used as a gene rank value *r* in GSEA to retain information about directionality of expression change on pathway level.

### Evaluation of Enrichment Algorithms

**FIGURE 1:**
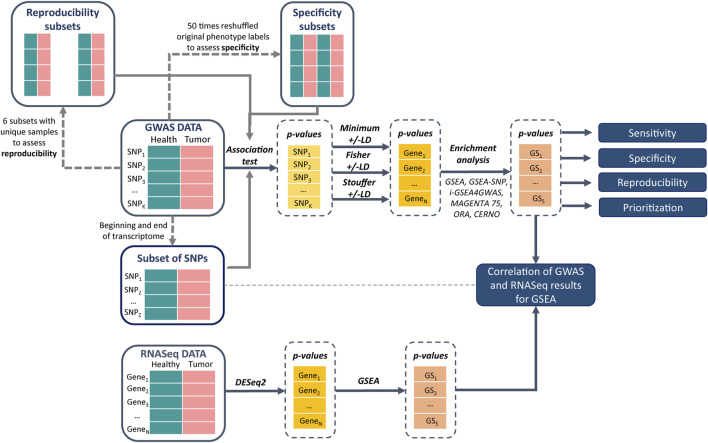
Pipeline of data analysis and gene set analysis (GSA) method evaluation.

The brief evaluation pipeline is presented in Figure 1. All described gene set enrichment algorithms were run with three different integration approaches (minimum, Fisher, and Stouffer) and with and without dependency correction for LD. Four metrics were calculated to evaluate the algorithms: sensitivity, specificity, prioritization, and reproducibility (Zyla et al., 2019). Sensitivity represents detection of target gene sets for a particular phenotype. Specifically, gene set *p*-values are collected, and the proportion of truly alternative hypotheses (1 − $\hat{\pi }$ ) is calculated with Storey’s method (Storey, 2002). Prioritization represents median ranks of target pathways in all analyzed gene sets. Specificity represents deviation of mean false-positive rate (FPR, observed level) from 5% (expected level). Specifically, FPR is the proportion of significant gene sets (*p *< 5%) among 50 permutations of the original phenotype. Reproducibility is the area under the curve (AUC) from the function of common detected gene sets across five or six data sets of the same phenotype at different cutoffs (Zyla et al., 2019). All used metrics were previously applied in transcriptomic data GSA (Tarca et al., 2013; Zyla et al., 2017; Zyla et al., 2019) and are one of the gold standards in enrichment algorithm evaluation (Geistlinger et al., 2021; Xie et al., 2021).

To test the impact of LD dependency correction, the differences between each metric separately within one enrichment method and integration approach were calculated (e.g., sensitivity of ORA minimum integration with LD correction minus ORA minimum integration without LD correction). Next, the impact of integration was assessed. As the minimum approach is mostly used, we referred its results to the Fisher and Stouffer methods. Again, the difference between performance metrics were calculated, but for different integrations (e.g., sensitivity of ORA Fisher integration with or without LD minus ORA minimum integration with or without LD).

Finally, we investigated similarities and information transition of gene set analysis performed on SNP and RNASeq data. For this purpose, we selected SNPs located at the “5′ UTR and upstream region” (beginning of transcript), as well as the “3′ UTR and downstream” coding region (end of transcript). The results of association test for those SNPs were extracted and aggregated using different integration methods with and without LD correction (the same as previously). Next, only the GSEA algorithm was run, as it has a direct equivalent in transcriptome analysis. The GSEA algorithm for RNASeq can distinguish up- and downregulated pathways; thus, the Spearman rank correlation was calculated for target pathways between GWAS (different SNP locations in transcript) and RNASeq (up-/downregulation). For the results from “5′UTR and upstream” GWAS location and gene set downregulation on RNASeq, the positive correlation is expected as SNPs in this region should block further transcription and translation. Opposite results are expected for “3′ UTR and downstream” where only isoforms of transcript products should be observed (Robert and Pelletier, 2018).

At each step of the evaluation process, the Kyoto Encyclopedia of Genes and Genomes (KEGG) database (Kanehisa et al., 2017) was used as a gene set collection (accessed January 15, 2021). In total, 341 gene sets were analyzed. The 54 target gene sets for breast cancer were selected through the literature search, and their detailed description is presented in Supplementary Table S2.

### Implementation of Gene Set Analysis Algorithms for Single-Nucleotide Polymorphism Data Analysis

The implementation of all evaluated algorithms is provided in the R package *intGSASNP* (integrative GSA for SNP), created for the purpose of this study. This package includes R functions to run selected gene set algorithms (ORA, CERNO, MAGENTA, GSEA, *i*-GSEA4GWAS, and GSEA-SNP) on SNP data. All algorithms were implemented according to the description included in the original manuscripts. *intGSASNP* allows the user to adjust various function parameters depending on the experiment, such as type of integration method (minimum, Fisher, and Stouffer), multiple testing correction method, permutation method (by gene entrez or SNP), number of permutations, incorporation of LD correction, or the number of processing cores required for parallel computing. In addition, an example of a dataset with sample refSNP IDs, entrez IDs, and *p*-values has been provided. Source code and the package documentation are available freely to download on GitHub (https://github.com/ZAEDPolSl/intGSASNP).

## Results

At first, results of SNP association tests were transformed to gene level by using minimum, Fisher, and Stouffer methods with and without LD correction. Then these results were used in combination with different GSA algorithms, i.e., GSEA, *i*-GSEA4GWAS, GSEA-SNP, MAGENTA75, ORA, and CERNO, and the four evaluation metrics were established, i.e., sensitivity, specificity, prioritization, and reproducibility (Figure 1). Based on those metrics, the impact of integration and correction for LD and the overall performance of tested methods were examined. Detailed results are presented in Supplementary Figures S1 and Supplementary Table S3. The total number of significant pathways is presented in Supplementary Table S4.

### Single-Nucleotide Integration Results

The number of significant genes (*p* < 0.05) for each method is presented in Table 1, while the coverage between approaches is presented in Supplementary Figure S2. It can be observed that application of LD correction decreased the number of significant transcripts and more likely reduced false-positive outcomes. Over 50% of genes were common to all integration techniques (54.98% and 55.03% with and without LD correction, respectively). Fisher and minimum approach shared around 30% (32.23% and 29.83% with and without LD correction, respectively) significant genes, which may lead to further similar results of GSA. The association between minimum and Fisher methods is characterized by large correlations (Supplementary Table S5). This effect is expected as Fisher integration method is not robust to asymmetrical *p*-values, which result in stronger association assigned to genes, similar to that of the minimum method. Stouffer’s technique showed weaker correlation to the minimum and Fisher methods. Also, after using Stouffer, there are not many unique significant genes or genes shared with only one of the other integration methods (Supplementary Figure S2). Over 90% of genes (96.56 and 91.81% with and without LD correction, respectively) indicated by the Stouffer method were also significant for the minimum and Fisher methods.

**TABLE 1:** Number of significant genes after integration of single-nucleotide polymorphism (SNP) association test results to gene level.

Integration	Minimum		Fisher		Stouffer	
LD correction	No	Yes	No	Yes	No	Yes
# of genes	12,759	10,810	11,401	10,456	7,382	6,948

### Overall Performance of Gene Set Analysis Methods

**FIGURE 2:**
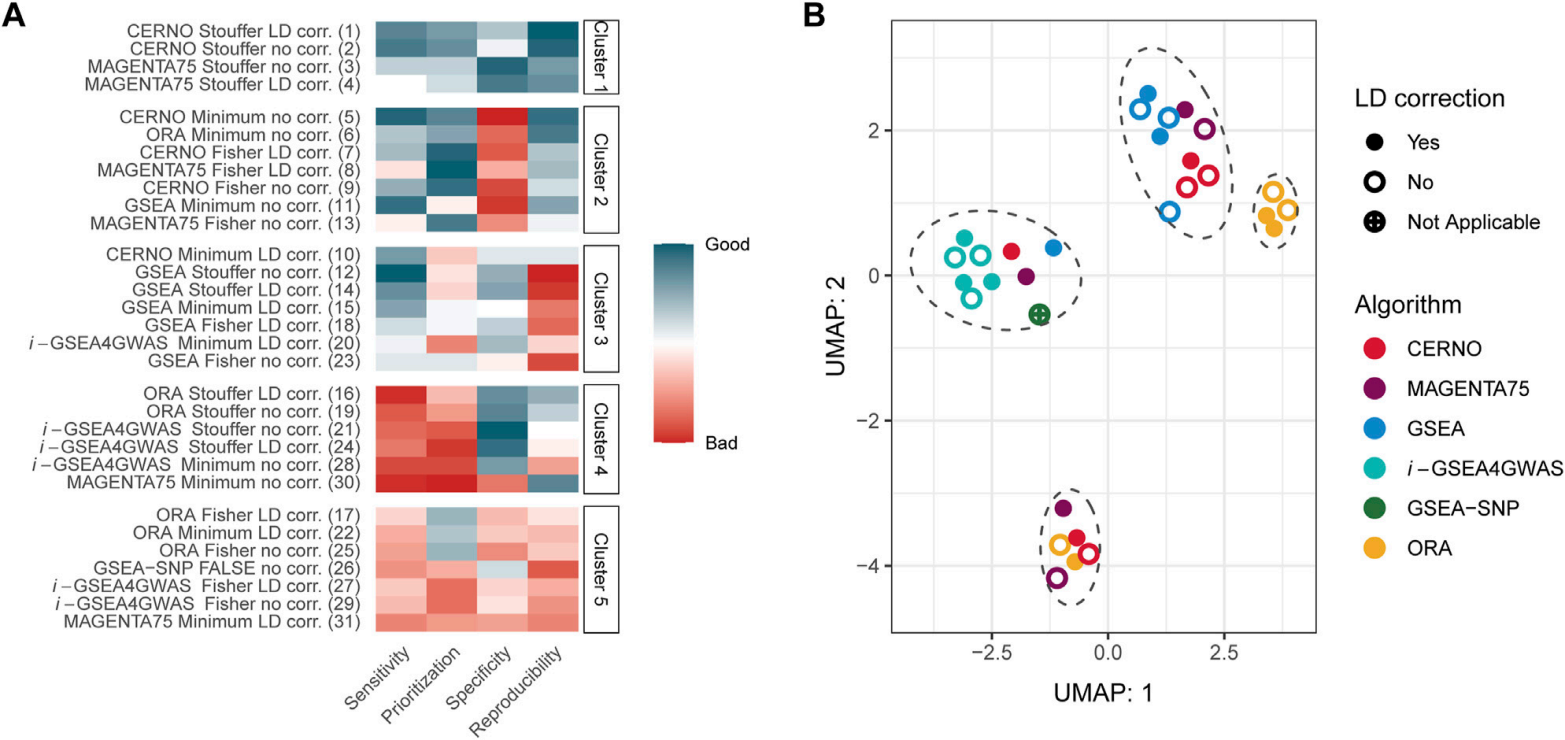
Overall evaluation of GSA algorithms. **(A)** Normalized evaluation metrics together with clustering results. The red color represents poor performance, while the blue color represents good performance. The number in the brackets, next to the algorithm name, illustrates the place in global ranking including all evaluation metrics. **(B)** UMAP projection for results of each algorithm and all 341 Kyoto Encyclopedia of Genes and Genomes (KEGG) pathways. Marked ellipses represent clustering within UMAP projection.

Within each evaluation metric, values were first normalized giving the lowest value for the best outcome and the highest for the worst. Next, the sum of all metrics was calculated, and algorithms were ranked from the best to the worst within the study. At last, results were clustered using the k-means approach, where the number of clusters were set by the Silhouette metric (optimal *k* equals 5). The best performance was obtained for CERNO and MAGENTA75 methods with Stouffer integration regardless of LD correction (Figure 2A, cluster 1). The worst outcomes were achieved for *i*-GSEA4GWAS and ORA with Fisher integration (regardless of LD correction) as well as for ORA and MAGENTA75 with minimum integration and LD correction and GSEA-SNP (cluster 5). Original GSEA gave moderate results in comparison with others (cluster 2 or 3). Overall, the results for CERNO and MAGENTA75 were the best (mostly in clusters 1 and 2), while *i*-GSEA4GWAS and ORA were the worst (mostly in clusters 4 and 5).

Next, global similarities of results were investigated by using the UMAP dimensionality reduction technique (McInnes et al., 2018; McInnes and Healy, 2018) on the GSA results for all 341 KEGG pathways (Figure 2B **)**. Four major clusters could be distinguished on two first instances of UMAP (Figure 2B). *i*-GSEA4GWAS gave similar results regardless of the integration technique as well as incorporation of LD correction. GSEA-SNP, CERNO, MAGENTA75, and GSEA performed on minimum integration and correction for LD are clustered together with *i*-GSEA4GWAS. The middle right cluster includes ORA with Fisher and minimum integration methods regardless of LD correction (color coding of UMAP projection due to integration used is presented in Supplementary Figure S3). Moreover, ORA with Stouffer integration gave similar results across all tested pathways to CERNO and MAGENTA75 (with the same integration method; bottom cluster).

### Impact of Linkage Disequilibrium Dependency Correction

**FIGURE 3:**
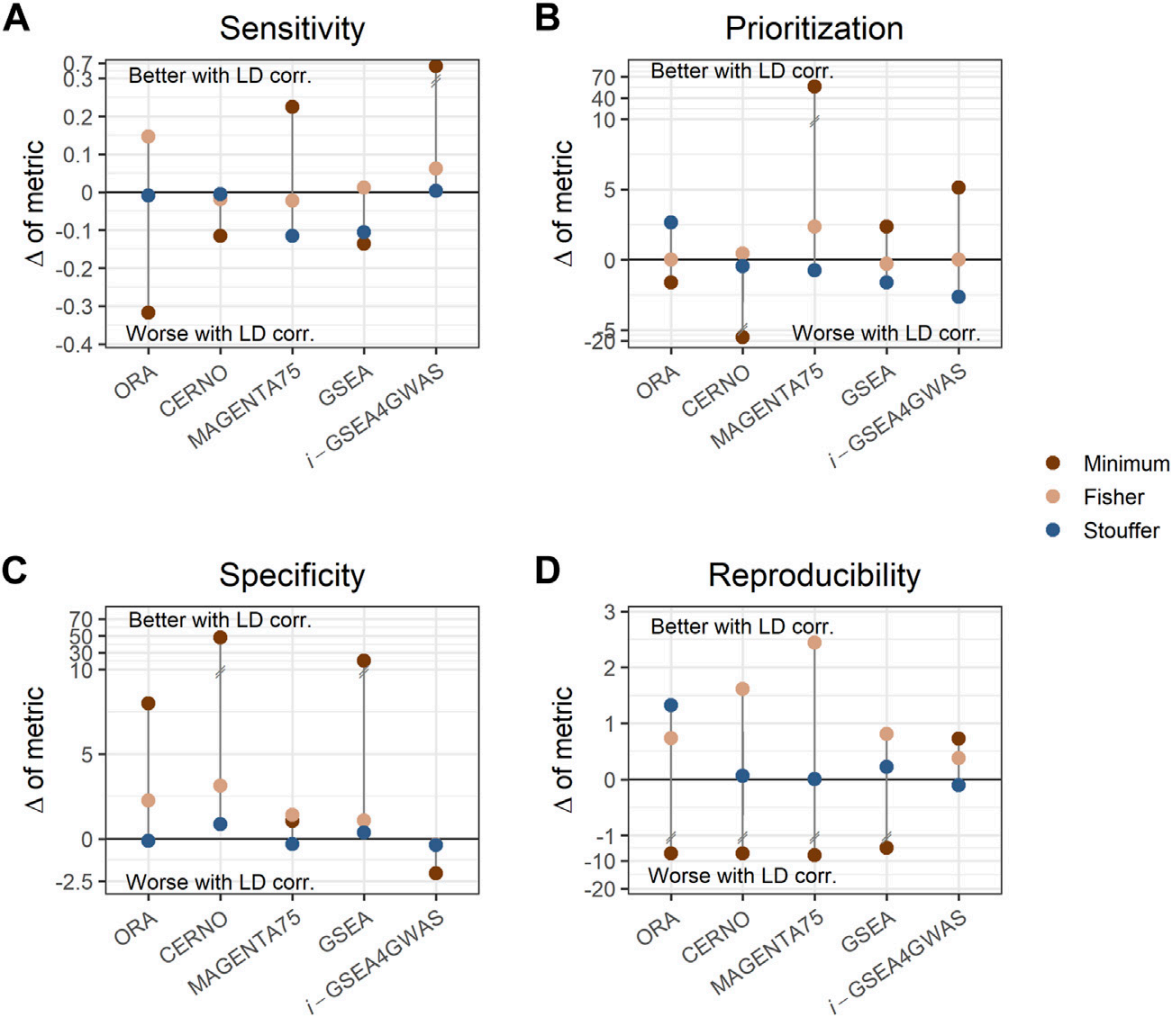
Impact of linkage disequilibrium (LD) correction on evaluation metrics. Each panel shows different metrics, i.e., sensitivity **(A)**, prioritization **(B)**, specificity **(C)**, and reproducibility **(D)**. Each dot represents the difference of metric when LD adjustment is used within a particular algorithm and integration method. Dots above the solid, black line represent better performance when LD correction is applied, while dots below the line represent the opposite. Colors show different integration techniques.

To investigate the impact of LD dependency correction, the difference of each evaluation metric within a particular algorithm performed with a specific integration method was calculated (e.g., sensitivity difference between ORA with minimum integration and LD correction, and ORA with minimum integration and without LD correction). Values of these differences are presented in Supplementary Table S6. ORA, CERNO, and GSEA showed decreased sensitivity (Figure 3A) when LD correction was applied, but the specificity was increased greatly (Figure 3C). The LD correction has a positive impact also on prioritization for MAGENTA75 (Figure 3B). *i*-GSEA4GWAS with Stouffer and Fisher integration gave similar performance regardless of LD correction usage. However, for minimum integration (default option in original implementation of the algorithm), the LD correction increases the sensitivity of the observed results with only a slight drop of specificity. For the remaining algorithms, when the Stouffer or Fisher integration method is used, the LD correction gave similar or better performance. When minimum integration is applied, the reproducibility decreases with LD correction (Figure 3D).

### Impact of *p*-Value Integration

**FIGURE 4:**
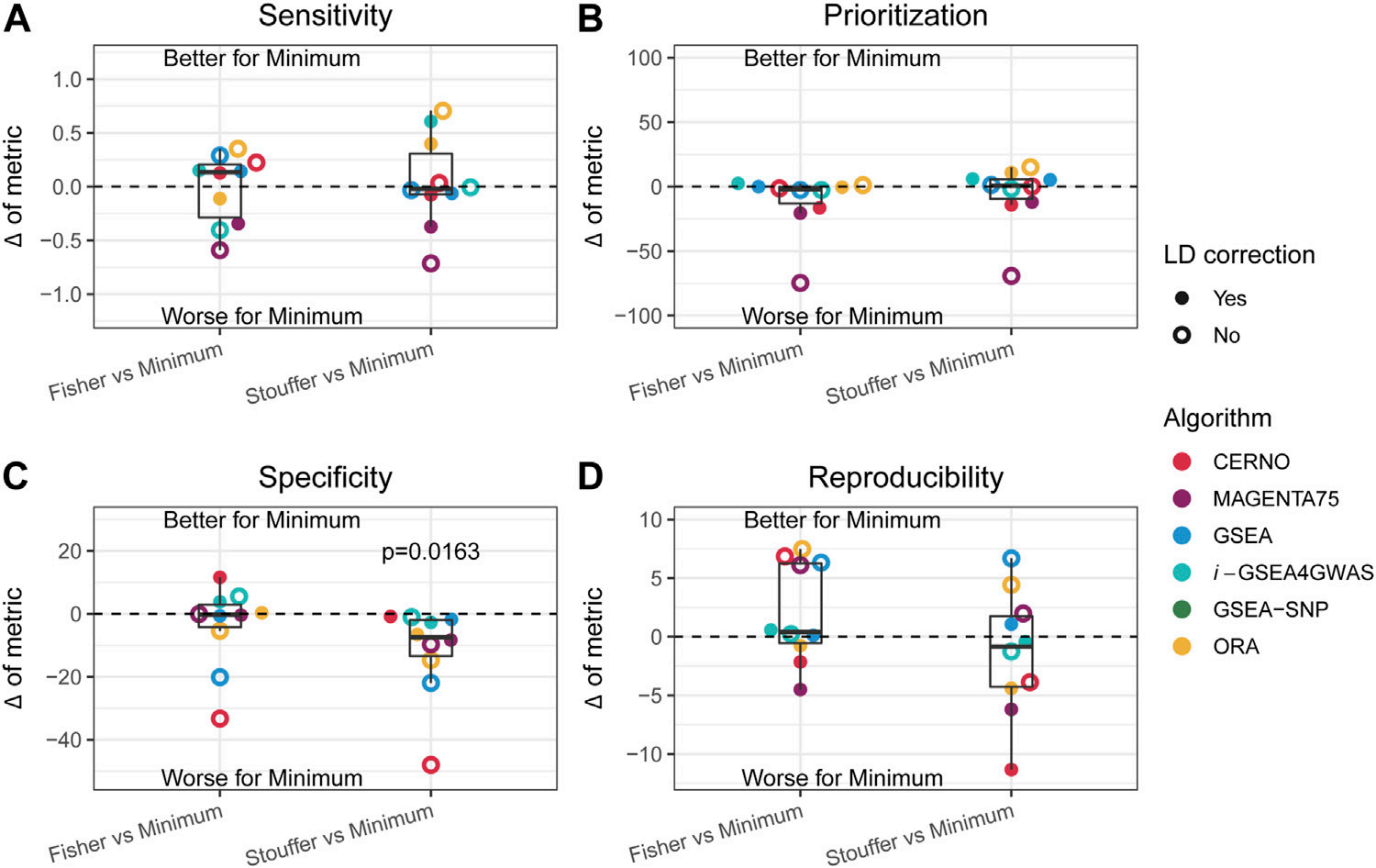
Impact of the Fisher and Stouffer integration technique compared with the minimum approach. **(A–D)** Differences between outcomes for the sensitivity, prioritization, specificity, and reproducibility, respectively. Colors represent different algorithms, and point shape represents whether correction for LD was applied. Dots above the solid, black line represent better performance for the minimum integration approach, while dots below the line represent the opposite.

As the minimum integration is the most preferred approach, the results of this aggregation technique were compared to those of the Fisher and Stouffer methods separately. For this purpose, the difference between performance metrics was calculated (e.g., sensitivity of ORA minimum integration with LD minus ORA Fisher integration with LD). In the previous paragraph, it was shown that LD correction has a beneficial impact in most of the cases, and it preserves biological insights, so further description will concentrate only on outcomes when dependency correction is applied. CERNO and MAGENTA75 had better results in terms of prioritization, specificity, and reproducibility when the Fisher or Stouffer method was used (Figure 4). *i*-GSEA4GWAS showed similar results regardless of the integration method used, with slightly better performance for minimum integration. The ORA algorithm gave similar performance when the minimum or Fisher method was applied, while Stouffer gave better specificity and reproducibility, but decreased sensitivity. Finally, GSEA showed better specificity when both Fisher and Stouffer were used, whereas other evaluation metrics were similar despite the integration techniques used (Figure 4).

Within each evaluation metric and obtained differences, the equivalence of mean to zero was tested by one-sample *t*-test. The Stouffer integration method gave significantly better results (*p*-value = 0.0163) in terms of specificity compared with minimum integration for all tested algorithms (Figure 4C). There was no statistically significant difference in other comparisons; however, the variety of effects can be observed for individual algorithms.

### Comparison of Gene Set Analysis on Single-Nucleotide and Gene Expression Level

As the enrichment methods were initially designed for transcriptome data analysis, target pathway similarities of outcomes observed for genome and transcriptome were investigated (Supplementary Figure S4). The same samples were taken for both omics, and the GSEA algorithm was applied (In RNASeq, it can distinguish up- and downregulated pathways). Moreover, for genomic data, only SNPs from the beginning and the end of transcriptomic regions were selected to catch the regulation directionality. Finally, the Spearman rank correlation coefficient was calculated between −log10(*p*-value_GeneSet_) from RNASeq and genomic data within pathways up- and downregulated separately.

**FIGURE 5:**
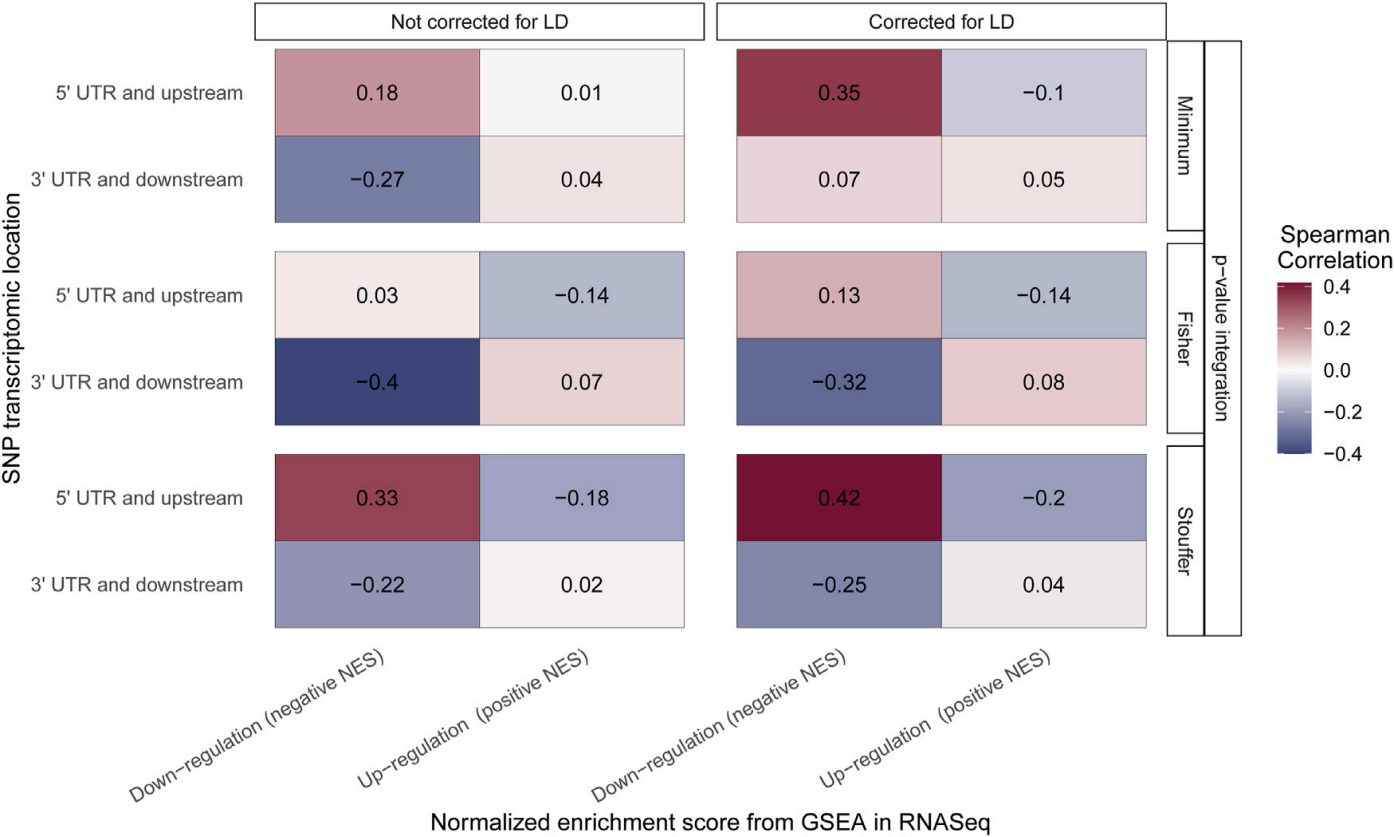
Correlation of the Gene Set Enrichment Analysis (GSEA) algorithm results performed on RNASeq and genomic data. The color of the boxes represents the value of Spearman rank correlation coefficient. The *x*-axis corresponds to the results from the GSEA algorithm performed on RNASeq data with distinction of pathway regulation direction. The *y*-axis corresponds to the results from the GSEA algorithm performed on genomic data with distinction of location of single-nucleotide polymorphisms (SNPs) taken to the aggregation process. All results are grouped by integration technique and LD correction used.

For Stouffer integration, the highest correlation of downregulated pathways and “5′ UTR and upstream” SNPs is observed, and it increases when LD correction is applied (from 0.33 to 0.42; both medium effect size) (Figure 5). Similar results can be observed for the minimum approach, where correlation changes from small effect size when no LD correction is applied to medium effect size with LD correction (from 0.18 to 0.35). For Fisher integration, the small effect size was observed only when LD adjustment was applied (Figure 5). When SNPs from “3′ UTR and downstream” (end of transcriptomic region) were analyzed, positive correlation with upregulated pathways was expected, but none of the tested methods showed statistically significant association. Nevertheless, for downregulated pathways, the expected negative correlation is observed for all integration techniques regardless of LD correction.

### Comparison of Gene Set Enrichment Analysis and Other Algorithms on Single-Nucleotide Polymorphism Level

Most of the tested algorithms were created by modification of the original GSEA method. Also, we found a correlation between GSEA results on genomic and transcriptomic level. Thus, we wanted to check how the results of GSA for target pathways are correlated between GSEA and other tested enrichment algorithms in GWAS (Supplementary Figure S5 **)**. The GSEA-SNP does not use integration in the process of enrichment analysis; nevertheless, it showed a small correlation with GSEA only when the Stouffer method is applied. On the other hand, the results of *i*-GSEA4GWAS had negative correlation with GSEA when the Fisher and Stouffer methods were applied. All other methods mostly showed positive correlation with GSEA. The highest correlation was observed for the CERNO and MAGENTA75 algorithms.

## Discussion

Incorporation of specific integration methods and LD correction can have significant impact on the performance of gene set analysis in GWAS. Usage of LD correction was beneficial for *i*-GSEA4GWAS, especially when the default minimum integration method was used. Thus, the incorporation of basic SNP dependency correction method, like we did here, or more complex solutions (de Leeuw et al., 2015) is recommended. When Fisher and Stouffer integration were used in the CERNO, ORA, MAGENTA75, and GSEA algorithms, the LD correction was always beneficial, so it should be applied in any case there. Observed decrease in reproducibility after applying LD could be the effect of decreasing the number of significant findings (genes with *p*-values lower than a threshold), which is usual after using correction for multiple testing (like LD correction here). Moreover, the reproducibility experiment was performed on much smaller subsets (*n* = 14 paired samples), which also decreased the power of GSA (Maleki et al., 2019). In *i*-GSEA4GWAS, this effect was not observed due to corrections given by SPES statistic.

Comparing *p*-value integration methods, Stouffer gave the best results in terms of specificity for all tested enrichment methods. Moreover, it gave better or similar results in terms of prioritization and reproducibility for CERNO, MAGENTA, GSEA, and ORA. The Stouffer integration decreases only sensitivity, which is an effect of preserving robustness to asymmetrical *p*-value distribution during the process of integration. Thus, the integrated *p*-value is higher, and some target pathways could not be detected. However, this mechanism prevents *p*-value overestimation that was observed for some GSA methods (Zyla et al., 2019). Also, Stouffer integration gave the highest correlation between GSA analysis results on SNP level and transcriptome level. Thus, this is the method that we recommend most.

SNPs located at the beginning of the gene region have the biggest ability to silence gene expression (Robert and Pelletier, 2018). The GSA outcomes compared between genomic and transcriptomic levels confirmed this effect. Furthermore, results for 5′ UTR and upstream SNPs were negatively correlated with upregulated pathways on the gene level. GSA results for SNPs located at the end of genes were positively correlated with upregulated pathways on the gene level, as it was expected, but the effect was smaller.

All gene set analysis methods, integration approaches, and LD correction method that were tested within the study were implemented in the *intGSASNP* R package and are freely available on GitHub. Therefore, different combinations of methods could be easily tested on any dataset by other researchers. We hope that collecting multiple methods in a single package will help to promote the application of GSA methods in SNP analysis.

In summary, we thoroughly analyzed different methods of gene set analysis in GWAS in terms of performance and its applicability. We showed that LD correction and Stouffer integration could increase the performance of enrichment analysis and encourage the introduction of these techniques into common practice. We believe that this work will guide others to select the most effective combinations of methods.

## Data Availability

Publicly available datasets were analyzed in this study. These data can be found here: https://www.cancer.gov/tcga.
