# Supplementary material for: Importance of SNP Dependency Correction and Association Integration for Gene Set Analysis in Genome-Wide Association Studies
Source: Front Genet. 2021 Dec 9;12:767358. doi: 10.3389/fgene.2021.767358 (PMC8696167; doi:10.3389/fgene.2021.767358)
Supplement: Supplementary file 1 [file DataSheet1.PDF]

Supplementary Material

1. Supplementary Figures

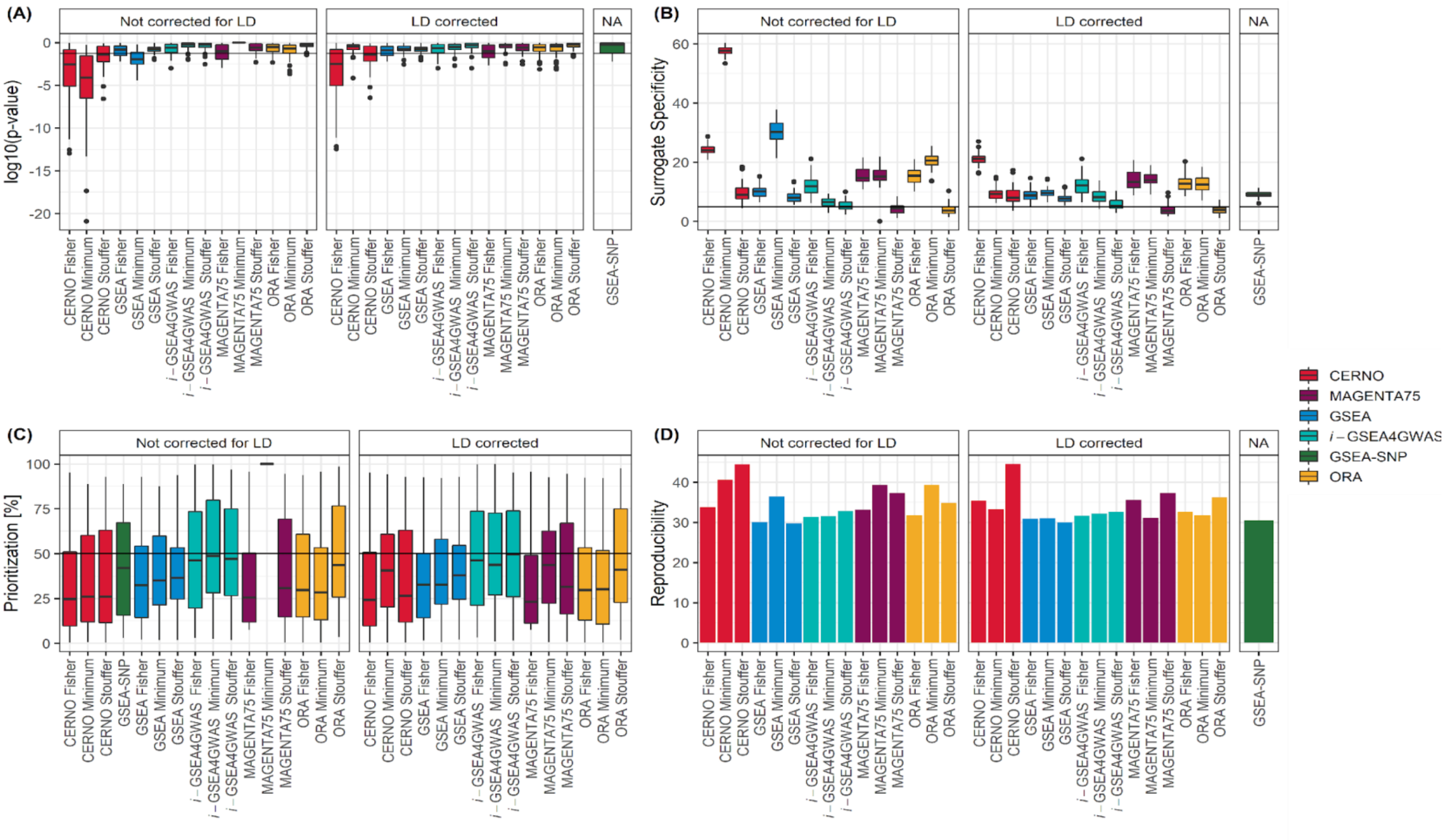

Supplementary Figure 1. Detailed results of GSA methods evaluation. Four metrics are presented: surrogate sensitivity, i.e. -log10(p-values) of target pathways (A); surrogate specificity, i.e. mean proportion of significant findings (p<5%) for 50 time phenotype permutation (B); surrogate prioritization, i.e. rank of target pathways (C); reproducibility as AUC (D).

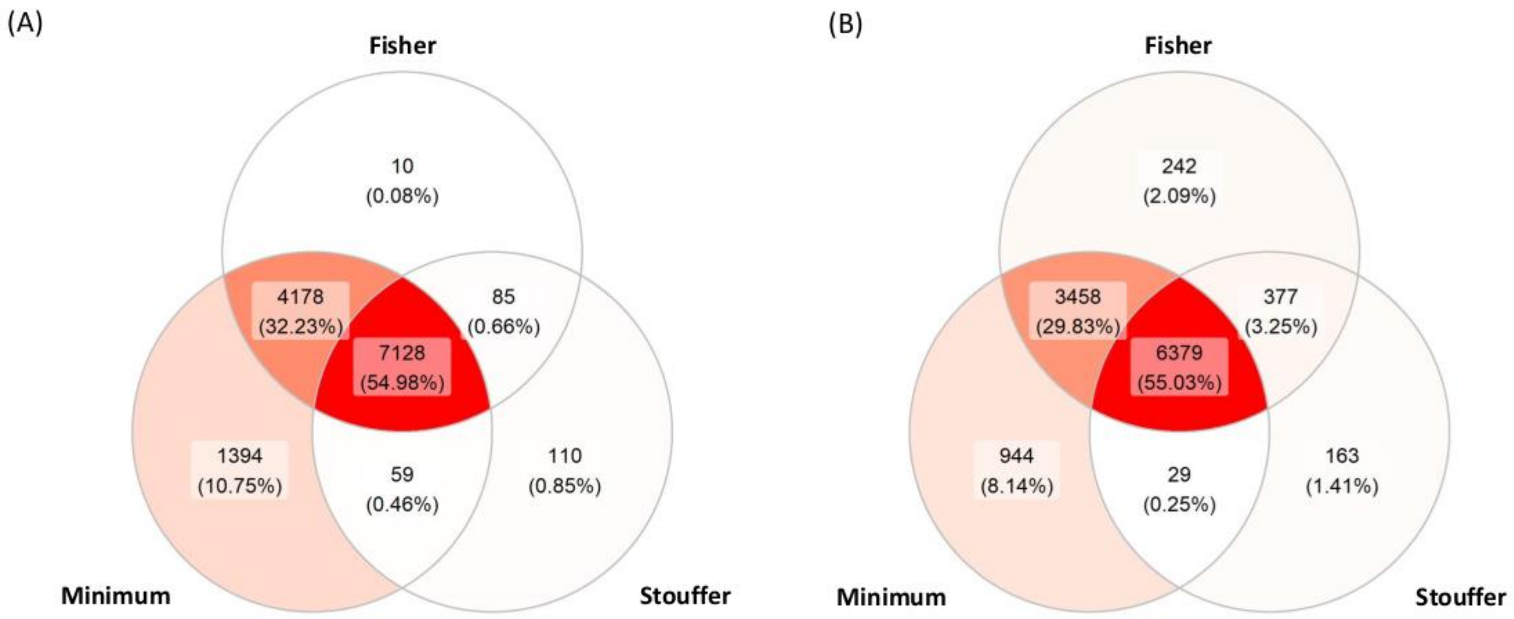

Supplementary Figure 2. Venn diagrams for significant genes (p-value<0.05) after using different methods of SNPs integration to gene level. Panel (A) shows results without LD correction, while panel (B) when LD correction was applied.

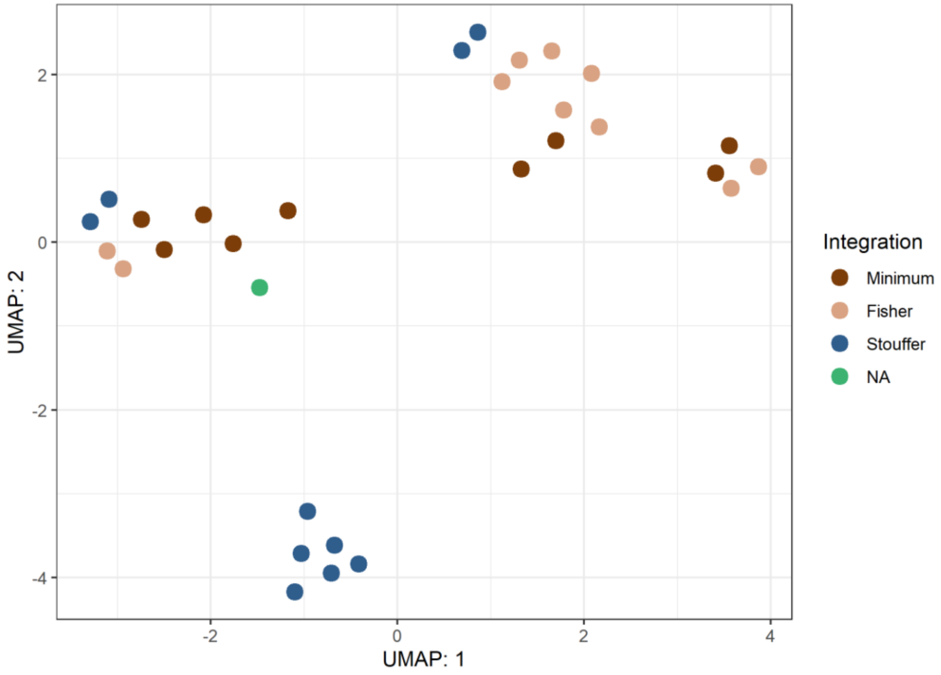

Supplementary Figure 3. UMAP projection for results of each algorithm and all analysed pathways. Colour represents different integration techniques.

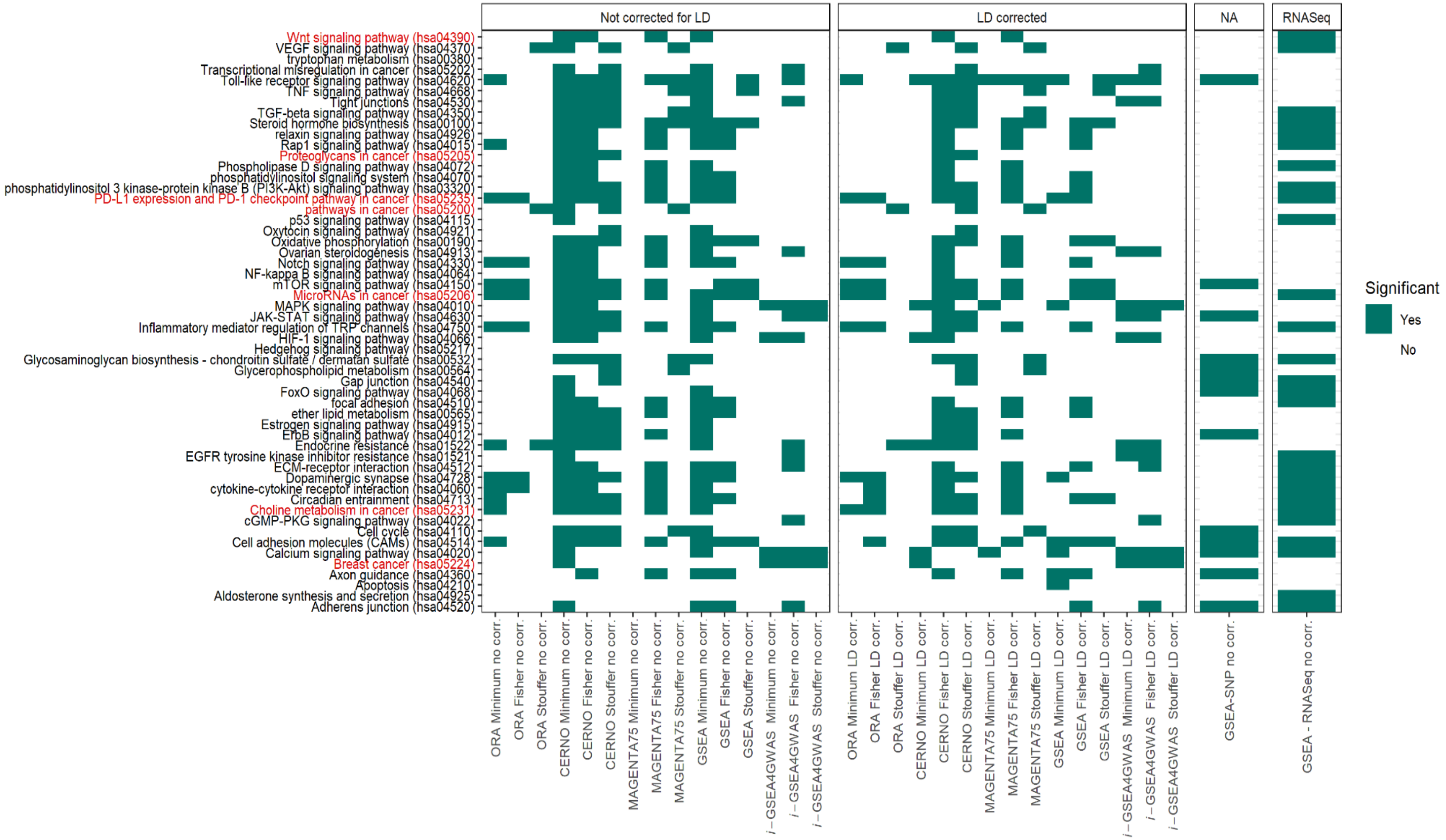

Supplementary Figure 4. Detection of breast cancer target pathways using different methods on SNP and mRNA level.

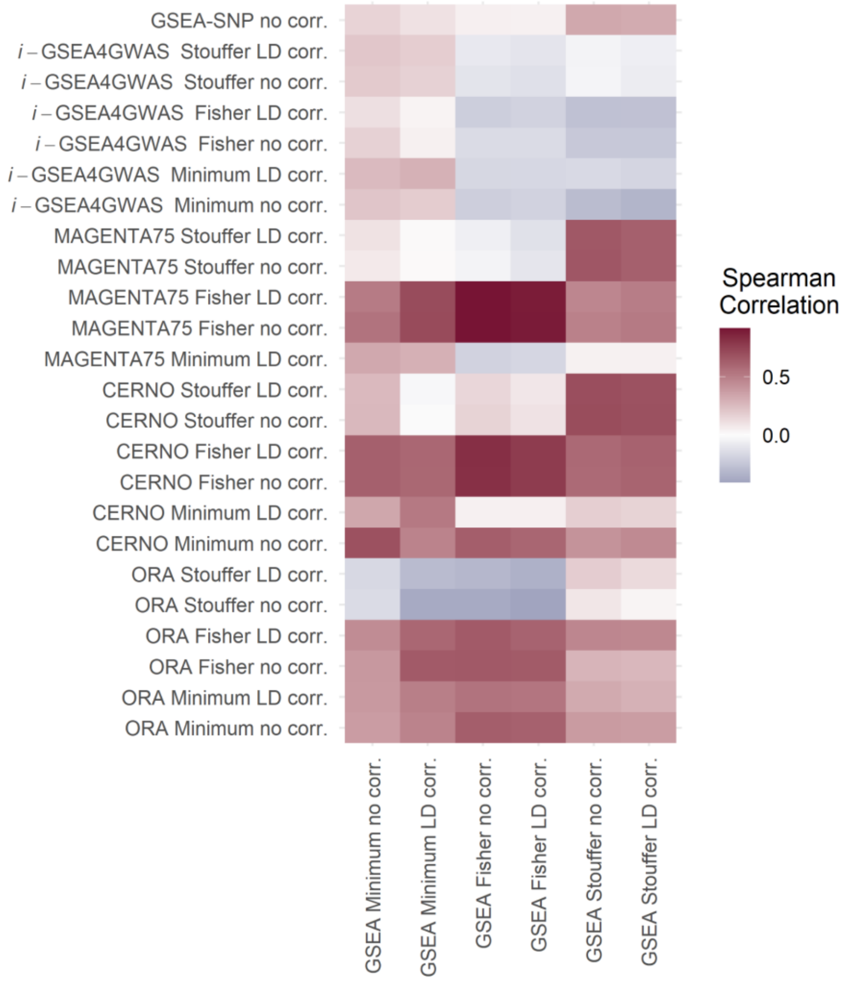

Supplementary Figure 5. Correlation of results for target pathways between GSEA and other tested algorithms on SNP level.

2. Supplementary Tables

Supplementary Table 1. Summary of breast cancer subtypes with regard to source center and patient ethnicity.

	**BREAST CANCER SUBTYPE**			**ALL**
	**HER2+**	**HR+**	**TNBC**	
	(**n** / % of N)	(**n** / % of N)	(**n** / % of N)	(**N** / % of *N.ALL*)
**ALL**	**16** / 19.28%	**55** / 66.27%	**12** / 14.46%	*N.ALL* = **83** / *100%*
**Ethnicity**				
**NOT HISPANIC OR LATINO**	**15** / 24.59%	**36** / 59.02%	**10** / 16.39%	**61** / *73.49%*
**NOT REPORTED**	**1** / 4.55%	**19** / 86.36%	**2** / 9.09%	**22** / *26.51%*
**Tissue Source Site**				
**ABS - IUPUI**	**0** / 0%	**1** / 100%	**0** / 0%	**1** / *1.20%*
**Christiana Healthcare**	**0** / 0%	**5** / 71.43%	**2** / 28.57%	**7** / *8.43%*
**International Genomics Consortium**	**3** / 100%	**0** / 0%	**0** / 0%	**3** / *3.61%*
**Roswell Park**	**0** / 0%	**4** / 66.67%	**2** / 33.33%	**6** / *7.23%*
**University of Pittsburgh**	**13** / 19.70%	**45** / 68.18%	**8** / 12.12%	**66** / *79.52%*

Supplementary Table 2. The detailed description of 54 selected target pathways for breast cancer from the KEGG database.

ID	Pathway	# of genes	PMID	Citation
hsa00100	Steroid hormone biosynthesis	20	26969590; 28527781	Capper, C. P., et al. (2016); Africander, D., and Storbeck, K. H. (2018).
hsa00190	Oxidative phosphorylation	133	29420223; 22134189; 29120753; 20849810	Ashton, T. M., et al. (2018); Whitaker-Menezes, D., et al. (2011); Zacksenhaus, E., et al. (2017); Solaini, G., et al. (2011).
hsa00380	Tryptophan metabolism	42	22444239; 31337401	Juhász, C., et al. (2012); Onesti, C. E., et al. (2019).
hsa00532	Glycosaminoglycan biosynthesis - chondroitin sulfate / dermatan sulfate	20	21658254; 22333131	Cooney, C. A., et al. (2011); Afratis, N., et al. (2012).
hsa00564	Glycerophospholipid metabolism	98	21222647; 28399876; 31819192	Dolce, V., et al. (2011); Luo, X., et al. (2017).; Koundouros, N., and Poulogiannis, G. (2020).
hsa00565	Ether lipid metabolism	49	23980144;	Benjamin, D. I., et al. (2013).
hsa01521	EGFR tyrosine kinase inhibitor resistance	79	18060492; 17549382	Normanno, N., et al. (2008); Ferrer-Soler, L., et al. (2007).
hsa01522	Endocrine resistance	98	32289273; 26455641; 29910644	Hanker, A. B., et al. (2020); Clarke, R., et al. (2015);

				AlFakeeh, A., and Brezden-Masley, C. (2018).
hsa03320	Phosphatidylinositol 3 kinase-protein kinase B (PI3K-Akt) signaling pathway	76	33375317; 29568883; 29417298	Miricescu, D., et al. (2021);<br>Du, L., et al. (2018);<br>Costa, R. L., et al. (2018).
hsa04010	MAPK signaling pathway	294	18060492; 31575382; 31746365; 29041978	Normanno, N., et al. (2008);<br>Chang, Y. S., et al. (2020);<br>Wu, X., Chen, S., and Lu, C. (2020);<br>Peng, W. X., et al. (2017).
hsa04012	ErbB signaling pathway	85	23000897	Koboldt, D. C. F. R., et al. (2012).
hsa04015	Rap1 signaling pathway	210	32906721	Looi, C. K., et al. (2020).
hsa04020	Calcium signaling pathway	240	31783287	Makena, M. R., and Rao, R. (2020).
hsa04022	cGMP-PKG signaling pathway	167	21777390; 25928539;	Fallahian, F., et al. (2011);<br>Wen, H. C., et al. (2015).
hsa04060	Cytokine-cytokine receptor interaction	295	25068787; 28860951	Esquivel-Velázquez, M., et al. (2015);<br>Lee, M., and Rhee, I. (2017).
hsa04064	NF-kappa B signaling pathway	104	32013374; 25386819; 32319568	Khongthong, P., et al. (2019);<br>Wang, W., et al. (2015);<br>Wang, X., et al. (2020).
hsa04066	HIF-1 signaling pathway	109	24156323; 25559953; 30943919; 33298946	Gilkes, D. M., and Semenza, G. L. (2013);<br>Liu, Z. J., et al. (2015);

				Campbell, E. J., et al. (2019); Ebright, R. Y., et al. (2020).
hsa04068	FoxO signaling pathway	131	26612860; 29440168; 26595803	Bullock, M. (2016); Hornsveld, M., et al. (2018); Smit, L., et al. (2016).
hsa04070	Phosphatidylinositol signaling system	97	21406469; 32276377; 11597319	Hernandez-Aya, L. F., and Gonzalez-Angulo, A. M. (2011); Owusu Obeng, E., et al. (2020); Fry, M. J. (2001).
hsa04072	Phospholipase D signaling pathway	148	23752189; 29145720	Henkels, K. M., et al. (2013); Cho, J. H., and Han, J. S. (2017).
hsa04110	Cell cycle	124	10647931; 30078354	Hanahan, D., and Weinberg, R. A. (2000); Thu, K. L., et al. (2018).
hsa04115	p53 signaling pathway	73	10647931	Hanahan, D., and Weinberg, R. A. (2000).
hsa04150	mTOR signaling pathway	155	33024820; 28400999	Cheng, T. Y. D., et al. (2020); Hare, S. H., and Harvey, A. J. (2017).
hsa04210	Apoptosis	136	21943236; 25824729; 10647931	Wong, R. S. (2011); Goldar, S., et al. (2015); Hanahan, D., and Weinberg, R. A. (2000).
hsa04330	Notch signaling pathway	53	31326555; 30111989; 32636747; 33034033	Krishna, B. M., et al. (2019); Kontomanolis, E. N., et al. (2018); Bai, J. W., et al. (2020);

				Shen, Q., and Reedijk, M. (2021).
hsa04350	TGF-beta signaling pathway	94	23022998; 17261761;	De Kruijf, E. M., et al. (2013);<br>Buck, M. B., and Knabbe, C. (2006).
hsa04360	Axon guidance	182	16015381; 21818544	Chedotal, A., et al. (2005);<br>Harburg, G. C., and Hinck, L. (2011).
hsa04370	VEGF signaling pathway	59	29254177	Chen, X., et al. (2017).
hsa04390	Wnt signaling pathway	157	33234169; 33080952	Xu, X., et al. (2020);<br>Koni, M., et al. (2020).
hsa04510	Focal adhesion	201	30728047	Rigiracciolo, D. C., et al. (2019).
hsa04512	ECM-receptor interaction	88	30061226; 26743193	Yeh, M. H., et al. (2018);<br>Insua-Rodríguez, J., and Oskarsson, T. (2016).
hsa04514	Cell adhesion molecules (CAMs)	149	23175431; 21499686; 19909018	Saadatmand, S., et al. (2013);<br>Li, D. M., and Feng, Y. M. (2011);<br>Makrilia, N., et al. (2009).
hsa04520	Adherens junction	71	23450077; 26901232; 33163938;	Knights, A. J., et al. (2012);<br>Yu, Y., and Elble, R. C. (2016);<br>Bischoff, P., et al. (2020).
hsa04530	Tight junctions	169	19920867; 30728783	Brennan, K., et al. (2009);<br>Bhat, A. A., et al. (2019).
hsa04540	Gap junction	88		Wu, J. I., and Wang, L. H. (2019).;

			30642339; 32209448; 30622930	Sinha, G., et al. (2020); Sinyuk, M., et al. (2018).
hsa04620	Toll-like receptor signaling pathway	104	31789409; 23526132	Shi, S., et al. (2020); Ahmed, A., et al. (2013).
hsa04630	JAK-STAT signaling pathway	162	33290193	Shao, F., et al. (2021).
hsa04668	TNF signaling pathway	112	31900901; 28791816	Cruceriu, D., et al. (2020); Ma, Y., et al. (2017).
hsa04713	Circadian entrainment	97	32581213; 31282103; 28704935	Hadadi, E., et al. (2020); Alkhanjaf, A. A. M., et al. (2019); Chacolla-Huaringa, R., et al. (2017).
hsa04728	Dopaminergic synapse	132	26477316	Borcherding, D. C., et al. (2016).
hsa04750	Inflammatory mediator regulation of TRP channels	98	31681615; 29772843; 23253476	Bujak, J. K., et al. (2019); Fels, B., et al. (2018); Ouadid-Ahidouch, H., et al. (2013).
hsa04913	Ovarian steroidogenesis	51	26969590; 28527781	Capper, C. P., et alJ. (2016); Africander, D., and Storbeck, K. H. (2018).
hsa04915	Estrogen signaling pathway	138	16421368; 30471920	Yager, J. D., and Davidson, N. E. (2006); Kulkoyluoglu-Cotul, E., et al. (2019).
hsa04921	Oxytocin signaling pathway	154	32782397	Liu, H., et al. (2020).
hsa04925	Aldosterone synthesis and secretion	98	27600178; 26969590; 26646587	Caroccia, B., et al. (2016); Capper, C. P., et al. (2016); Rigiracciolo, D. C., et al. (2016).
hsa04926	Relaxin signaling pathway	129		Fue, M., et al. (2018);

			30126180; 14520686; 11147585	Silvertown, J. D., et al. (2003);<br>Hombach-Klonisch, S., et al. (2000).
hsa05200	Pathways in cancer	531		
hsa05202	Transcriptional misregulation in cancer	192		
hsa05205	Proteoglycans in cancer	205	25829250; 32984308; 17922662	Theocharis, A. D., et al. (2015);<br>Ahrens, T. D., et al. (2020);<br>Eshchenko, T. Y., et al. (2007).
hsa05206	MicroRNAs in cancer	310		
hsa05217	Hedgehog signaling pathway	63	31027259; 15342389; 23547970	Galdo, R. D., et al. (2019);<br>Kubo, M., et al. (2004);<br>Hui, M., et al. (2013).
hsa05224	Breast cancer	147		
hsa05231	Choline metabolism in cancer	98		Hilvo, M., and Matej Orešiè, A. (2012).
hsa05235	PD-L1 expression and PD-1 checkpoint pathway in cancer	89	28346916	Schütz, F., et al. (2017).

Supplementary Table 3. Summary of all gene set analysis evaluation metrics.

Method	# of significant target pathway	% of significant target pathway	Sensitivity (the higher the better)	Prioritization (the lower the better)	Specificity (the lower the better)	Reproducibility (the higher the better)
ORA Minimum	13	24.07	0.7151	28.45	15.62	39.32
ORA Minimum LD	8	14.81	0.3980	30.06	7.60	31.82
ORA Fisher	7	12.96	0.3628	29.62	10.22	31.84
ORA Fisher LD	10	18.52	0.5090	29.62	7.96	32.57
ORA Stouffer	3	5.56	0.0096	43.55	0.94	34.89
ORA Stouffer LD	3	5.56	0.0000	40.91	1.05	36.22
CERNO Minimum	46	85.19	0.9857	25.95	52.55	40.61
CERNO Minimum LD	6	11.11	0.8705	40.62	4.50	33.23
CERNO Fisher	37	68.52	0.7618	24.63	19.26	33.76
CERNO Fisher LD	37	68.52	0.7431	24.19	16.11	35.37
CERNO Stouffer	29	53.70	0.9517	26.10	4.54	44.49

**CERNO Stouffer LD**	29	53.70	0.9468	26.54	3.67	44.56
**MAGENTA75 Minimum**	0	0.00	0.0000	100.00	10.27	39.26
**MAGENTA75 Minimum LD**	3	5.56	0.2250	43.55	9.22	31.09
**MAGENTA75 Fisher**	25	46.30	0.5913	25.37	10.22	33.16
**MAGENTA75 Fisher LD**	25	46.30	0.5689	23.02	8.82	35.60
**MAGENTA75 Stouffer**	9	16.67	0.7138	30.79	0.59	37.28
**MAGENTA75 Stouffer LD**	9	16.67	0.5981	31.52	0.90	37.29
**GSEA Minimum**	39	72.22	0.9580	35.04	25.16	36.41
**GSEA Minimum LD**	8	14.81	0.8221	32.70	4.60	31.01
**GSEA Fisher**	19	35.19	0.6683	32.40	5.05	30.09
**GSEA Fisher LD**	18	33.33	0.6798	32.70	3.96	30.89
**GSEA Stouffer**	7	12.96	0.9908	36.36	3.21	29.72

**GSEA Stouffer LD**	8	14.81	0.8852	37.98	2.85	29.94
**iGSEA4GWAS Minimum**	4	7.41	0.0086	48.83	1.52	31.53
**iGSEA4GWAS Minimum LD**	10	18.52	0.6248	43.70	3.56	32.25
**iGSEA4GWAS Fisher**	14	25.93	0.4122	46.19	7.06	31.30
**iGSEA4GWAS Fisher LD**	14	25.93	0.4744	46.19	7.39	31.67
**iGSEA4GWAS Stouffer**	4	7.41	0.0152	47.07	0.48	32.78
**iGSEA4GWAS Stouffer LD**	3	5.56	0.0180	49.71	0.85	32.68
**GSEASNP**	13	24.07	0.2320	42.08	3.99	30.47

Supplementary Table 4. Number of significant pathways detected by each algorithm with different method of aggregation from SNP to gene level.

	ORA	CERNO	MAGENT75	GSEA	*i*-GSEA4GWAS	GSEA-SNP*
**Minimum**	41/22	231/26	0/15	193/46	28/44	47
**Fisher**	29/26	133/130	79/73	65/64	66/84	
**Stouffer**	15/12	100/98	27/30	43/43	22/22	

Number of significant pathways without LD correction / Number of significant pathways with LD correction.

* Integration and LD correction is not applicable.

Supplementary Table 5. Spearman rank correlation between integrated results of SNP-phenotype association.

Integration method	Minimum	Fisher	Stouffer
**Minimum**	1.00/1.00	0.90/0.85	0.28/0.41
**Fisher**	0.90/0.85	1.00/1.00	0.38/0.43
**Stouffer**	0.28/0.41	0.38/0.43	1.00/1.00

Without LD correction / With LD correction

Supplementary Table 6. Differences of each evaluation metric with and without LD correction with regard to algorithm and integration method.

Sensitivity					
	**ORA**	**CERNO**	**MAGENTA75**	**GSEA**	***i*-GSEA4GWAS**
**Minimum**	-0.32	-0.12	0.23	-0.14	0.62
**Fisher**	0.15	-0.02	-0.02	0.01	0.06
**Stouffer**	-0.01	0	-0.12	-0.11	0
**Prioritization**					
	**ORA**	**CERNO**	**MAGENTA75**	**GSEA**	***i*-GSEA4GWAS**
**Minimum**	-1.61	-14.66	56.45	2.35	5.13
**Fisher**	0	0.44	2.35	-0.29	0
**Stouffer**	2.64	-0.44	-0.73	-1.61	-2.64
**Specificity**					
	**ORA**	**CERNO**	**MAGENTA75**	**GSEA**	***i*-GSEA4GWAS**
**Minimum**	8.01	48.05	1.05	20.56	-2.04
**Fisher**	2.26	3.16	1.4	1.09	-0.33
**Stouffer**	-0.11	0.87	-0.31	0.36	-0.37
**Reproducibility**					
	**ORA**	**CERNO**	**MAGENTA75**	**GSEA**	***i*-GSEA4GWAS**
**Minimum**	-7.5	-7.39	-8.17	-5.4	0.72
**Fisher**	0.73	1.62	2.44	0.81	0.38
**Stouffer**	1.33	0.07	0	0.22	-0.11
